# Supplementary material for: Social Stress Increases Vulnerability to High-Fat Diet-Induced Insulin Resistance by Enhancing Neutrophil Elastase Activity in Adipose Tissue
Source: Cells. 2020 Apr 16;9(4):996. doi: 10.3390/cells9040996 (PMC7226953; doi:10.3390/cells9040996)
Supplement: Supplementary file 1 [file cells-09-00996-s001.zip › Motoyama Revised Supplementary data (Cells).docx]

**Supplementary Information**

**Social stress increases vulnerability to high-fat diet-induced insulin resistance by enhancing neutrophil elastase activity in adipose tissue**

Shinichiro Motoyama­^1^­, Hiroyuki Yamada^1^, Keita Yamamoto^1^, Noriyuki Wakana^1^,

Kensuke Terada^1^, Masakazu Kikai^1^, Naotoshi Wada^1^, Makoto Saburi^1^,

Takeshi Sugimoto^1^, Hiroshi Kubota^1^, Daisuke Miyawaki^1^, Daisuke Kami^2^, Takehiro Ogata^3^, Masakazu Ibi^4^, Chihiro Yabe-Nishimura^4^, Satoaki Matoba^1^

^1^Department of Cardiovascular Medicine, ^2^Department of Regenerative Medicine, ^3^Department of Pathology and Cell Regulation, ^4^Department of Pharmacology,

Graduate School of Medical Science,

Kyoto Prefectural University of Medicine, Kyoto, Japan

**Supplementary Figure Legends**

**Supplementary Fig. S1.** **Apparatuses for the exposure to social stress. (A)** Photographs of perforated partition. **(B)** Photograph of housed mice during exposure to social stress. Control, unstressed control mice; Stress, stressed mice.

**Supplementary Fig. S2.** **Schema of the design of the different experiments.**

**Supplementary Fig. S3.** **Effects of SS on body weight, epididymal white adipose tissue (eWAT) weight, and caloric intake. (A)** Body weight measurements were equivalent between the two groups. Values represent the mean ± SEM for 10 (Control) and 9 (Stress) mice. **(B)** eWAT weight and eWAT/body weight values were equivalent between the two groups. Values represent the mean ± SEM for 10 (Control) and 10 (Stress) mice. **(C)** Cumulative caloric intake over 6 weeks was equivalent between the two groups. Mice were group housed 3 mice per cage. Values represent the mean ± SEM for 3 (Control) and 3 (Stress) cages. Control, unstressed control mice; Stress, stressed mice.

**Supplementary Fig. S4.** **Effects of SS and RSD on glucose and insulin tolerance before HFD feeding. (A and B)** Glucose and insulin tolerance tests before HFD feeding. Values represent the mean ± SEM for 10 (Control), 9 (Stress), and 8 (Defeat) mice. Control, unstressed control mice; Stress, stressed mice; Defeat, repeated social defeated mice.

**Supplementary Fig. S5.** **Effects of SS on the percentage of peripheral blood monocytes after HFD feeding.** Flow cytometric analysis of CD11b and Ly-6C expression in lineage (B220, CD11c, NK1.1, CD49b, CD90.2, Ly-6G, F4/80, and I-Ab)-negative blood populations. Quantitative analysis showed no difference in peripheral blood Ly-6C monocytes fractions between the two groups. Values represent the mean ± SEM for 8 (Control) and 9 (Stress) mice. Control, unstressed control mice; Stress, stressed mice.

**Supplementary Fig. S6.** **Effects of SS on the fraction of eWAT macrophages.** **(A and B)** Flow cytometric analysis of CD45, F4/80, CD11b, CD11c, and CD206 expression in eWAT stromal vascular cells before HFD (A) and after HFD (B). Quantitative analysis showed no difference between the two groups. Values represent the mean ± SEM for 10 (Control) and 10 (Stress) mice before HFD as well as 10 (Control) and 10 (Stress) mice after HFD. Control, unstressed control mice; Stress, stressed mice.

**Supplementary Fig. S7. Quantitative RT-PCR analysis of mRNA expression levels in eWAT before and after HFD feeding.** Values represent the mean ± SEM relative to Control after HFD except for TNF-α(relative to Control before HFD). Each group consisted of 4 (Control before HFD), 4 (Stress before HFD), 9-10 (Control after HFD), and 8-10 (Stress after HFD) samples. **p* < 0.05 vs. Stress before HFD, ^#^*p* < 0.05 vs. Control after HFD. Control, unstressed control mice; Stress, stressed mice; HFD, high-fat diet.

**Supplementary Fig. S8.** **Neutrophil elastase (NE) activation in lower limbs after HFD feeding. (A and B)** Representative ex vivo images of lower limbs as well as quantitative measurement of radiant efficiency corresponding to NE activity. Values represent the mean ± SEM. Each group consisted of 10 (Control) and 10 (Stress) mice. Control, unstressed control mice; Stress, stressed mice; HFD, high-fat diet.

**Supplementary Fig. S9.** **Effects of SS on the number of peripheral blood neutrophils.** Flow cytometric analysis of CD11b and Ly-6G expression in lineage (B220, NK1.1, CD49b, CD90.2, Ter119, and CD115)-negative blood populations. Quantitative analysis showed no difference in peripheral blood Ly-6G/CD11b fractions between the two groups before and after HFD. Values represent the mean ± SEM for 5 (Control) and 5 (Stress) mice before HFD as well as 10 (Control) and 10 (Stress) mice after HFD. HFD, high-fat diet; Control, unstressed control mice; Stress, stressed mice.

**Supplementary Fig. S10.** **Effects of SS on the fraction of eWAT neutrophils after HFD.** **(A)** Flow cytometric analysis of CD11b and Ly-6G expression in lineage (F4/80 and CD11c)-negative eWAT stromal vascular cells after HFD. **(B)** Quantitative analysis showed no difference between the two groups. Values represent the mean ± SEM for 10 (Control) and 10 (Stress) mice. SSC, side-scattered light; FSC, forward-scattered light; PI, propidium iodide; eWAT, epididymal white adipose tissue; Control, unstressed control mice; Stress, stressed mice.
