# Supplementary material for: Social Stress Increases Vulnerability to High-Fat Diet-Induced Insulin Resistance by Enhancing Neutrophil Elastase Activity in Adipose Tissue
Source: Cells. 2020 Apr 16;9(4):996. doi: 10.3390/cells9040996 (PMC7226953; doi:10.3390/cells9040996)
Supplement: Supplementary file 1 [file cells-09-00996-s001.zip › Motoyama Revised Supplementary Figure (Cells).pptx]

## Slide 1
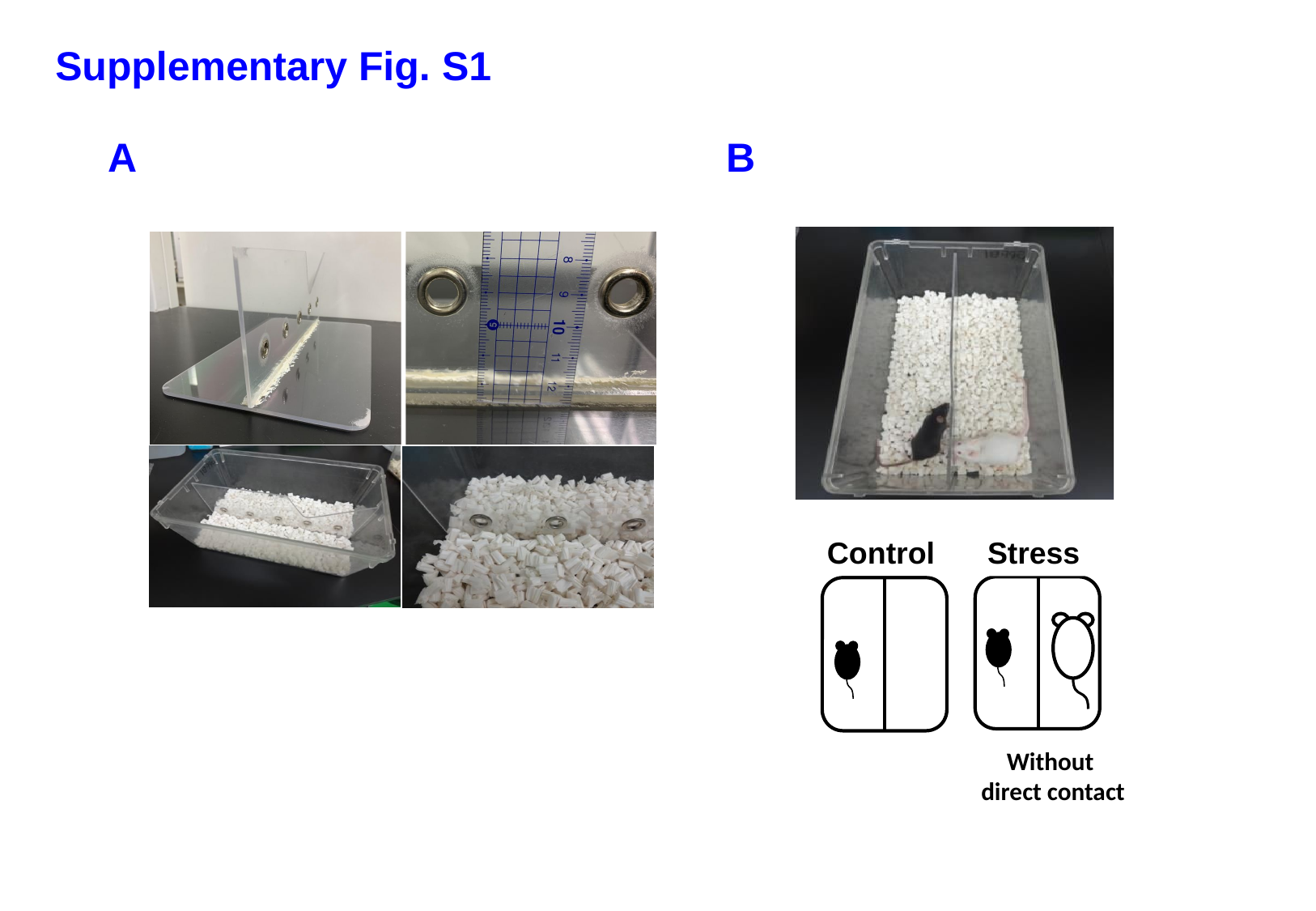

Supplementary Fig. S1
A
B
Control
Stress
Without direct contact

## Slide 2
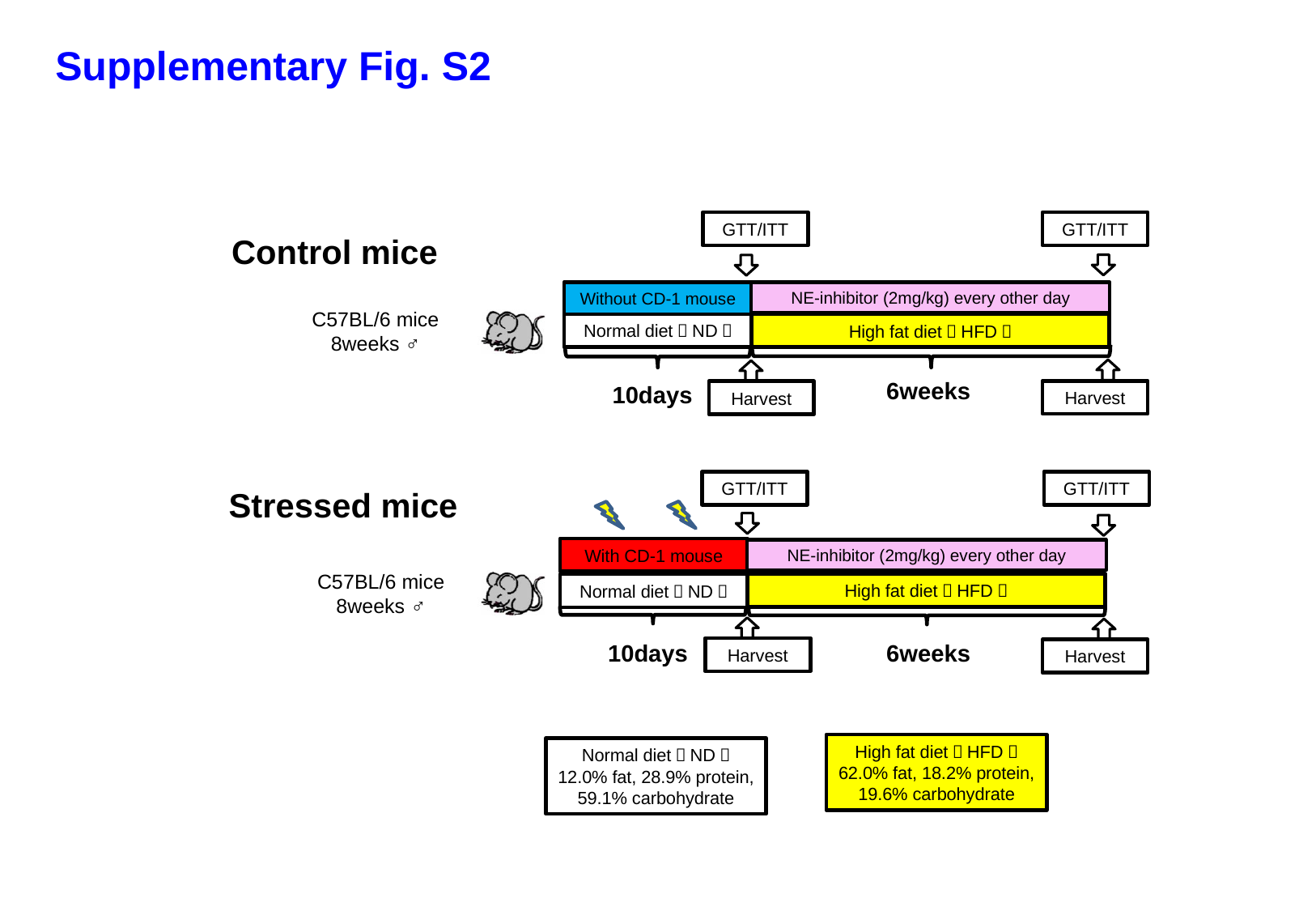

Supplementary Fig. S2
GTT/ITT
GTT/ITT
Control mice
Without CD-1 mouse
NE-inhibitor (2mg/kg) every other day
C57BL/6 mice
8weeks ♂
Normal diet（ND）
High fat diet（HFD）
6weeks
10days
Harvest
Harvest
GTT/ITT
GTT/ITT
Stressed mice
With CD-1 mouse
NE-inhibitor (2mg/kg) every other day
C57BL/6 mice
8weeks ♂
High fat diet（HFD）
Normal diet（ND）
6weeks
10days
Harvest
Harvest
High fat diet（HFD）
62.0% fat, 18.2% protein,
19.6% carbohydrate
Normal diet（ND）
12.0% fat, 28.9% protein,
59.1% carbohydrate

## Slide 3
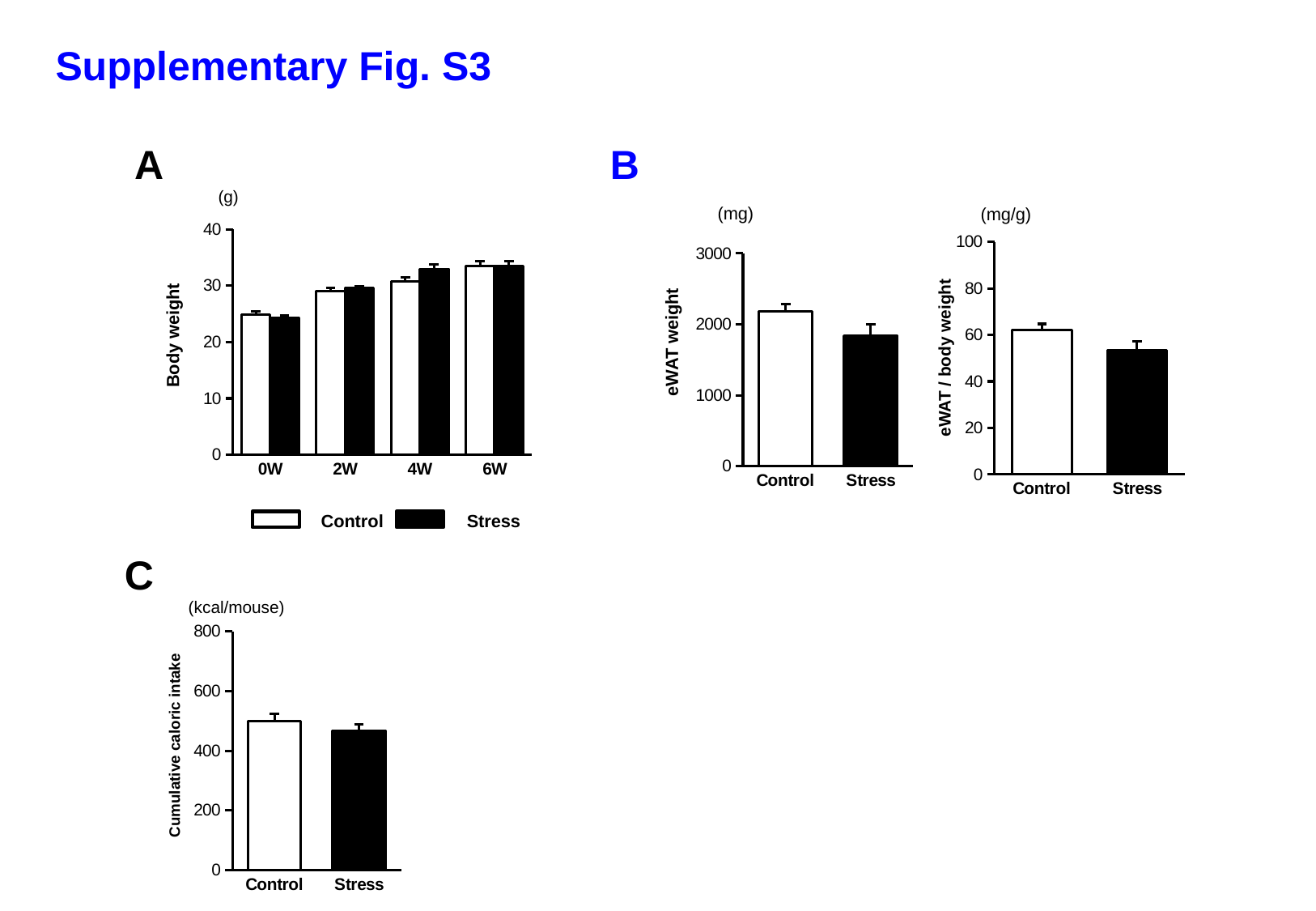

Supplementary Fig. S3
A
B
(g)
(mg)
(mg/g)
### Chart
| Category | Control | Stress |
|---|---|---|
| 0W | 24.89 | 24.222222222222218 |
| 2W | 29.05 | 29.566666666666663 |
| 4W | 30.790000000000003 | 32.97777777777778 |
| 6W | 33.510000000000005 | 33.5 |
### Chart
| Category | 平均値 |
|---|---|
| Control | 62.233241157272595 |
| Stress | 53.54368240901922 |
### Chart
| Category | 平均値 |
|---|---|
| Control | 2182.9 |
| Stress | 1833.8 |Body weight
eWAT weight
eWAT / body weight
Stress
Control
C
(kcal/mouse)
### Chart
| Category | 平均値 |
|---|---|
| Control | 499.7781333333333 |
| Stress | 467.0537 |Cumulative caloric intake

## Slide 4
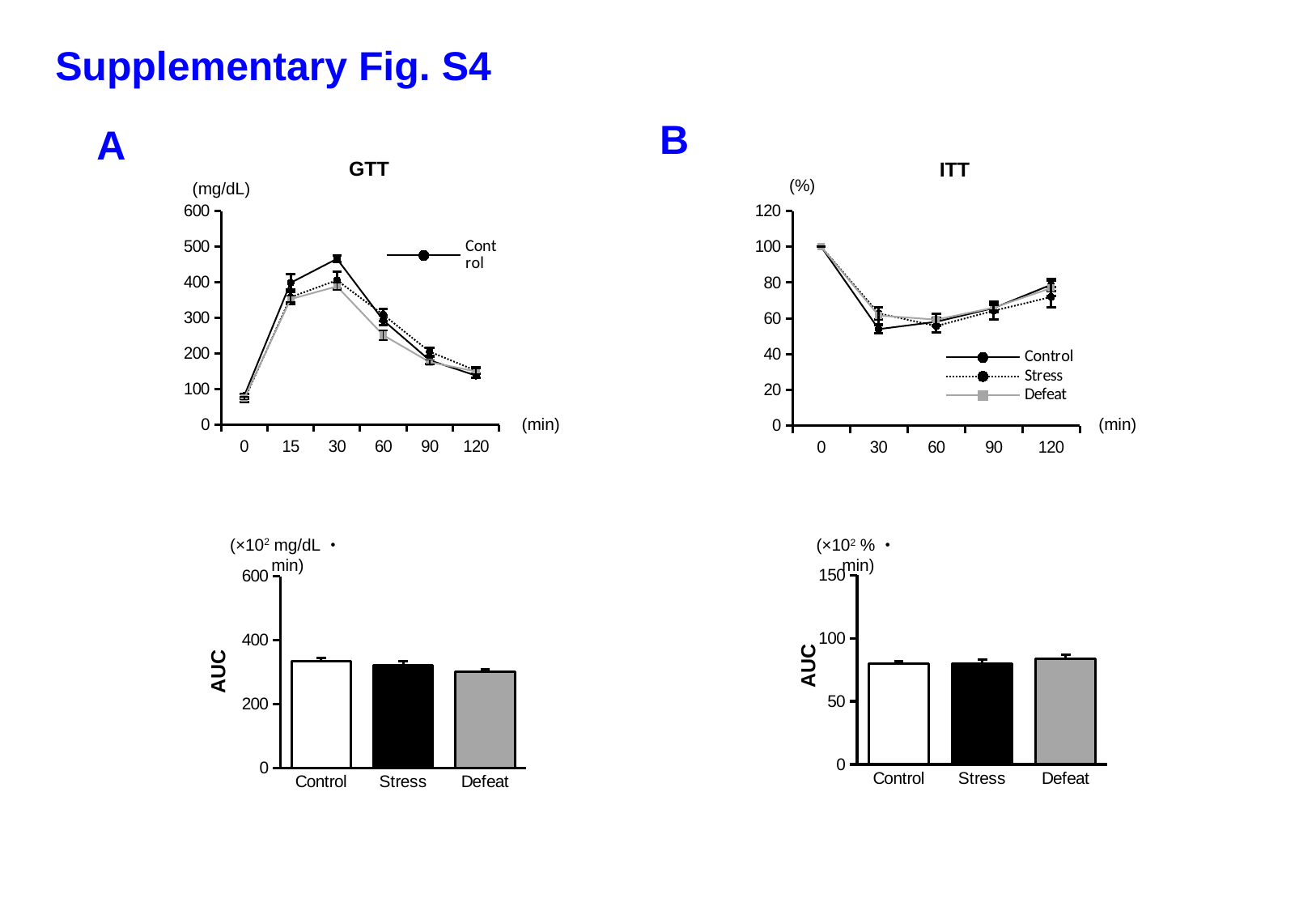

Supplementary Fig. S4
B
A
GTT
ITT
(%)
(mg/dL)
### Chart
| Category | Control | Stress | Defeat |
|---|---|---|---|
| 0 | 81.3 | 70.25 | 74.55555555555556 |
| 15 | 398.7 | 358.5 | 353.0 |
| 30 | 466.2 | 406.0 | 388.55555555555554 |
| 60 | 293.4 | 309.0 | 251.55555555555554 |
| 90 | 181.1 | 205.375 | 176.0 |
| 120 | 138.1 | 152.25 | 150.0 |
### Chart
| Category | Control | Stress | Defeat |
|---|---|---|---|
| 0 | 100.0 | 100.0 | 100.0 |
| 30 | 53.94618649098305 | 62.79573661350446 | 61.575495140898255 |
| 60 | 58.07796913383993 | 55.70517024655205 | 59.30838124001366 |
| 90 | 65.74857473413863 | 64.22170198550103 | 65.95394635584745 |
| 120 | 78.79099363215364 | 71.99330009408195 | 76.9274420960249 |(min)
(min)
(×102 mg/dL・min)
(×102 %・min)
### Chart
| Category | 平均値 |
|---|---|
| Control | 80.15046815251154 |
| Stress | 79.71983994206573 |
| Defeat | 83.89648988876904 |
### Chart
| Category | 平均値 |
|---|---|
| Control | 333.8625 |
| Stress | 320.1666666666667 |
| Defeat | 301.18125 |AUC
AUC

## Slide 5
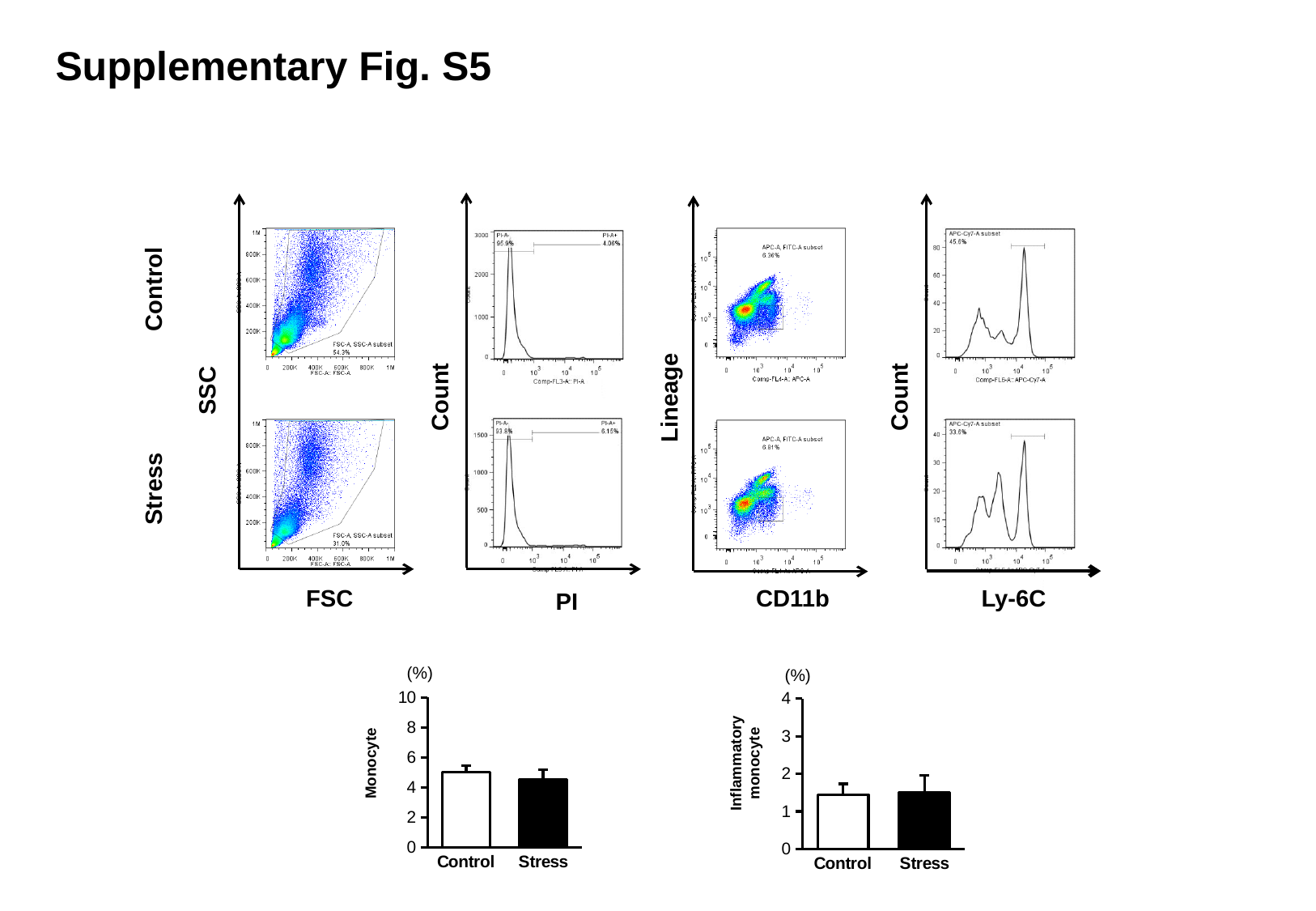

Supplementary Fig. S5
Control
SSC
Count
Count
Lineage
Stress
FSC
Ly-6C
CD11b
PI
(%)
(%)
### Chart
| Category | 平均値 |
|---|---|
| Control | 5.0225 |
| Stress | 4.561111111111111 |
### Chart
| Category | 平均値 |
|---|---|
| Control | 1.447605325 |
| Stress | 1.5096151111111111 |Inflammatory monocyte
Monocyte

## Slide 6
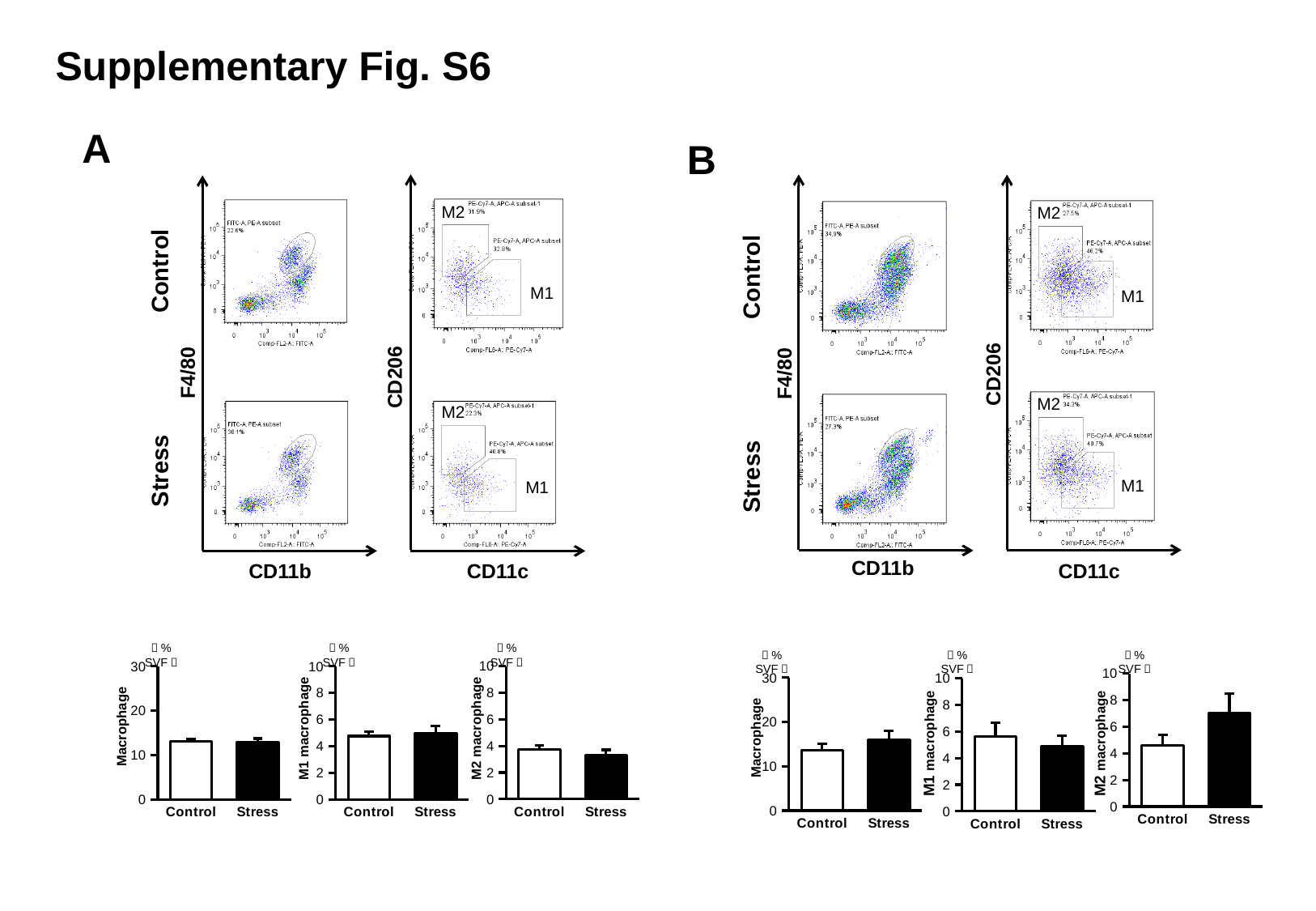

Supplementary Fig. S6
A
B
M2
M2
Control
Control
M1
M1
F4/80
F4/80
CD206
CD206
M2
M2
Stress
Stress
M1
M1
CD11b
CD11b
CD11c
CD11c
（% SVF）
（% SVF）
（% SVF）
（% SVF）
（% SVF）
（% SVF）
### Chart
| Category | 平均値 |
|---|---|
| Control | 3.7540000000000004 |
| Stress | 3.2900000000000005 |
### Chart
| Category | 平均値 |
|---|---|
| Control | 13.02 |
| Stress | 12.949000000000002 |
### Chart
| Category | 平均値 |
|---|---|
| Control | 4.752000000000001 |
| Stress | 4.945 |
### Chart
| Category | 平均値 |
|---|---|
| Control | 4.619999999999999 |
| Stress | 7.0 |
### Chart
| Category | 平均値 |
|---|---|
| Control | 13.55 |
| Stress | 15.969999999999999 |
### Chart
| Category | 平均値 |
|---|---|
| Control | 5.646 |
| Stress | 4.911999999999999 | Macrophage
M1 macrophage
M2 macrophage
 Macrophage
M1 macrophage
M2 macrophage

## Slide 7
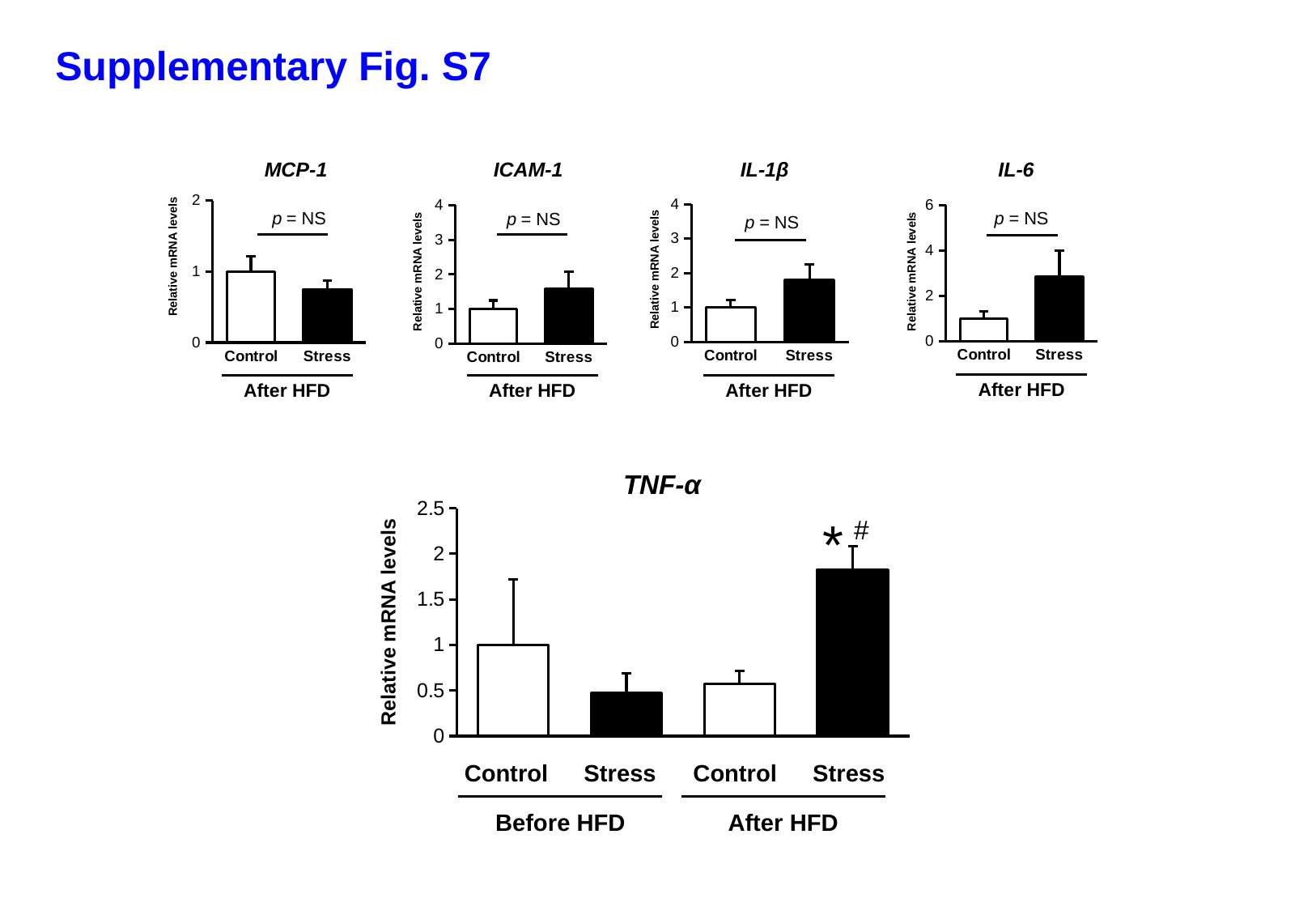

Supplementary Fig. S7
MCP-1
ICAM-1
IL-1β
IL-6
### Chart
| Category | 平均値 |
|---|---|
| Control | 0.9965510587567885 |
| Stress | 1.813534385241205 |
### Chart
| Category | 平均値 |
|---|---|
| Control | 1.0014322594701768 |
| Stress | 0.7465351265113794 |
### Chart
| Category | 平均値 |
|---|---|
| Control | 0.996433466197128 |
| Stress | 1.5850957642244041 |
### Chart
| Category | 平均値 |
|---|---|
| Control | 0.998541472818115 |
| Stress | 2.8511298245957883 |p = NS
p = NS
p = NS
p = NS
Relative mRNA levels
Relative mRNA levels
Relative mRNA levels
Relative mRNA levels
After HFD
After HFD
After HFD
After HFD
TNF-α
### Chart
| Category | |
|---|---|#
*
Relative mRNA levels
Control
Stress
Control
Stress
Before HFD
After HFD

## Slide 8
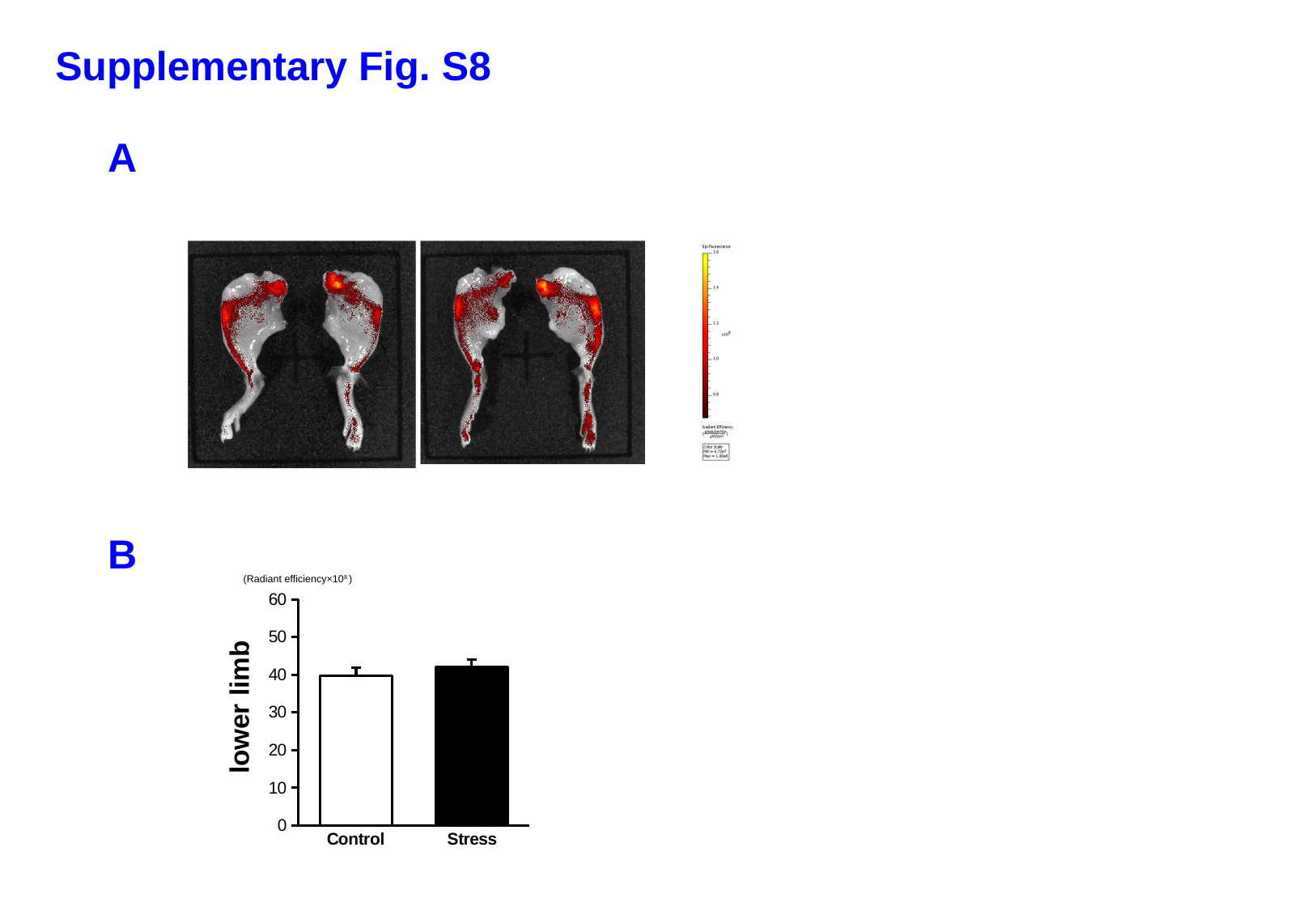

Supplementary Fig. S8
A
B
(Radiant efficiency×108 )
### Chart
| Category | 平均値 |
|---|---|
| Control | 39.826 |
| Stress | 42.146 |lower limb

## Slide 9
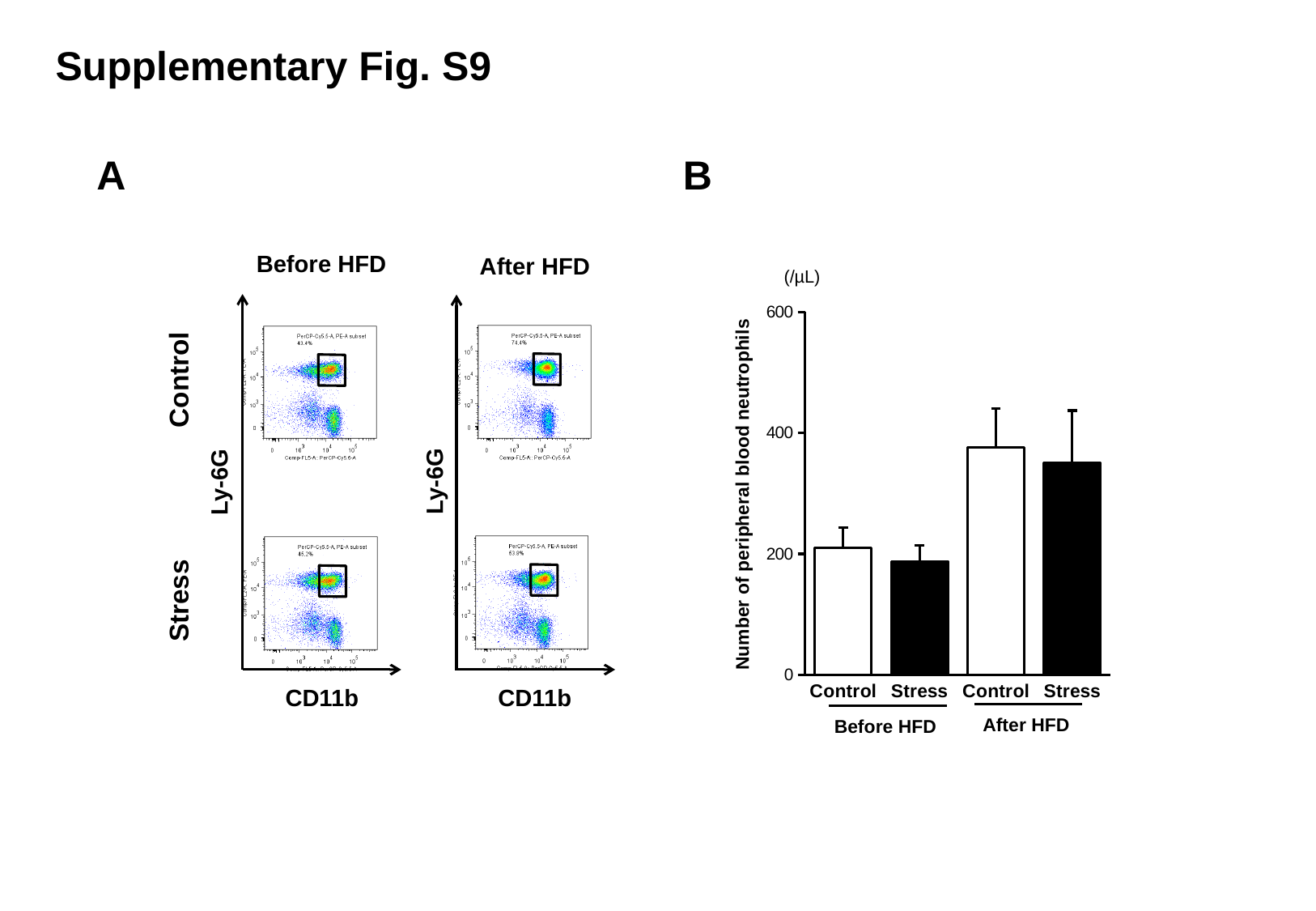

Supplementary Fig. S9
B
A
Before HFD
After HFD
(/µL)
### Chart
| Category | 平均値 |
|---|---|
| Control | 210.13400000000001 |
| Stress | 187.656 |
| Control | 375.677 |
| Stress | 349.965 |
Control
Ly-6G
Ly-6G
Number of peripheral blood neutrophils
Stress
CD11b
CD11b
After HFD
Before HFD

## Slide 10
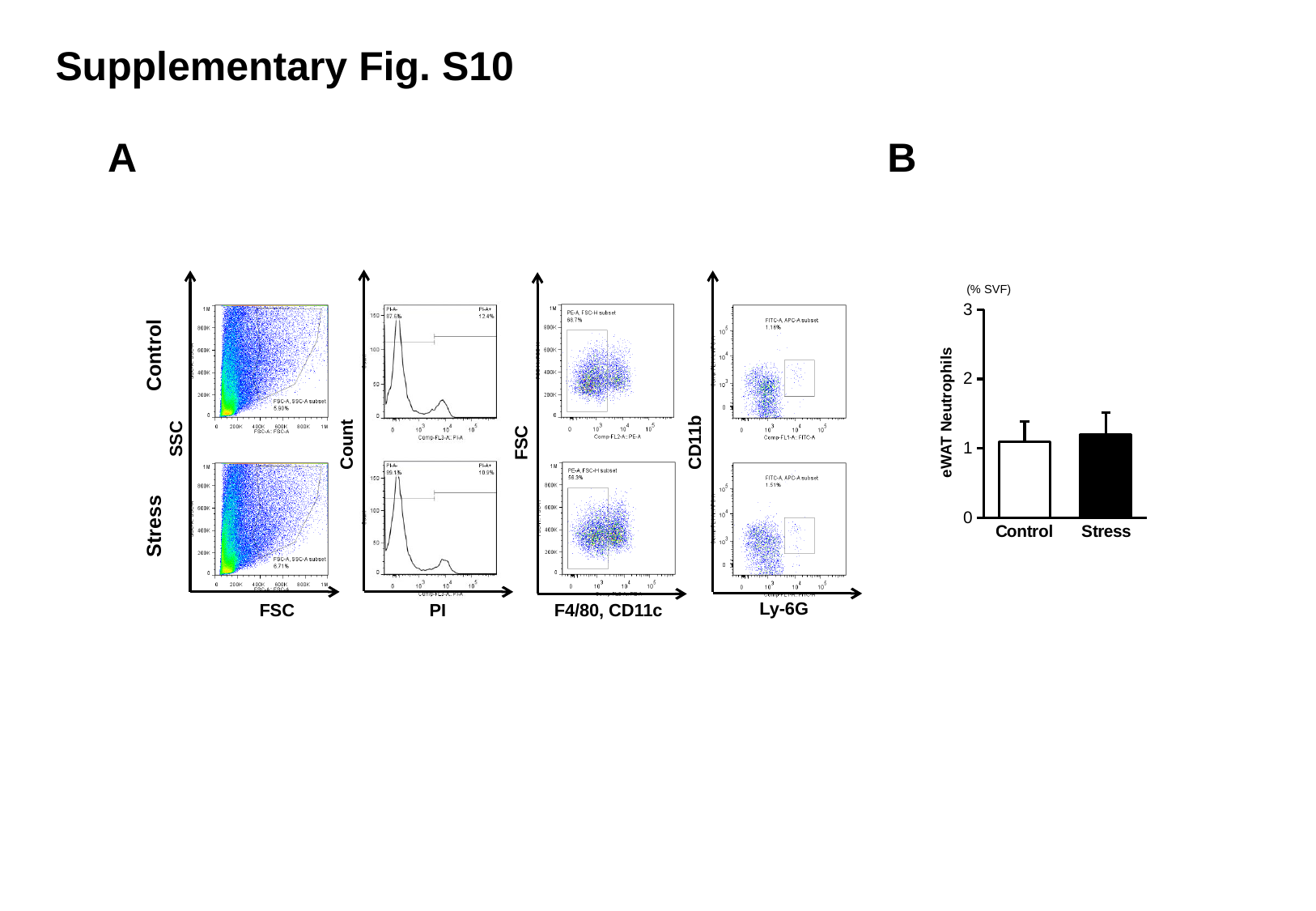

Supplementary Fig. S10
A
B
(% SVF)
### Chart
| Category | 平均値 |
|---|---|
| Control | 1.0950000000000002 |
| Stress | 1.198 |Control
eWAT Neutrophils
SSC
CD11b
FSC
Count
Stress
Ly-6G
F4/80, CD11c
FSC
PI
